# Editorial: Vitamin D and COVID-19: New Mechanistic and Therapeutic Insights

**DOI:** 10.3389/fphar.2022.882046

**Published:** 2022-03-15

**Authors:** Ewa Marcinkowska, Geoffrey Brown

**Affiliations:** ^1^ Department of Biotechnology, University of Wroclaw, Wroclaw, Poland; ^2^ School of Biomedical Sciences, Institute of Clinical Sciences, University of Birmingham, Birmingham, United Kingdom

**Keywords:** vitamin D, COVID-19, deficiency, cytokine storm, immunity

A PubMed search using the terms “vitamin D and COVID-19” reveals more than 1,000 papers. Mostly they indicate a correlation between a low level of vitamin D and a severe outcome from COVID-19 disease (Grant et al., 2022). However, it is easy and quite common in medicine to confound causality with a simple correlation. Moreover, such confusion is particularly plausible for vitamin D research. The problem arises from naming vitamin D a “vitamin,” whereas, in fact, it is a hormone that is produced when the human skin is exposed to sunlight. Therefore, vitamin D is more likely to be produced in people who are fit enough to spend time outdoors. Consequently, a proper blood level of vitamin D is much more likely in fit and healthy people than in sick people. The best means of differentiating a random correlation from causal inference is to describe the underlying mechanism (Pearl and Mackenzie, 2019).

The active metabolite of vitamin D is the steroid hormone 1,25-dihydroxyvitamin D (1,25D) (Carlberg, 2014a). Vitamin D is produced from 7-dehydrocholesterol when the human skin is exposed to UV light. Then, vitamin D activation occurs in two steps: 25-hydroxylation followed by 1α-hydroxylation (Prosser and Jones, 2004). The first step occurs in the liver, where vitamin D undergoes hydroxylation to 25-hydroxyvitamin D (25D). 25D undergoes hydroxylation in the kidneys to the highly active metabolite 1,25D. Both 25D and 1,25D circulate in the blood *via* the vitamin D binding protein (DBP). 25D bound to DBP has a long half-life of around 2–3 weeks, whereas 1,25D has a short half-life of 10–20 h (Carter, 2011). Hence, serum 25D concentration is used routinely to measure the vitamin D status in the human body (Sempos et al., 2012). Hydroxylation of 1,25D at carbon atom C-24, catalyzed by 24-hydroxylase of 1,25D (CYP24A1), is the first step of its inactivation. A view is that the CYP24A1 level is downregulated by sex hormones and that a higher level of 17β-estradiol may enhance the actions of 1,25D. Peruzzu et al. presented data to support this mechanism.

Vitamin D deficiency is perhaps the most common nutritional deficiency in the world. Recently, there has been increasing awareness of vitamin D deficiency, and its supplementation is now much more common, but seldom regularly controlled. Regarding vitamin D deficiency, the currently agreed levels of serum 25D are deficiency at < 12 ng/ml, insufficiency at 12–20 ng/ml, and levels between 20 and 50 ng/ml are considered to be adequate. In their article, Hafez et al. reported that vitamin D deficiency does correlate significantly with higher mortality from COVID-19.

The receptor for 1,25D is the vitamin D receptor (VDR), which, upon binding to the ligand, translocates from the cytoplasm to the cell nucleus. There, VDR acts as a ligand-activated transcription factor to regulate the transcription of its target genes (Aranda and Pascual, 2001). Hundreds of genes are regulated by VDR (Pike and Meyer, 2014); many of them are responsible for maintaining calcium-phosphate homeostasis (Holick, 1996). However, it is also well known that 1,25D regulates genes involved in the function of immune cells, for example, CD14, which is the macrophage co-receptor for bacterial lipopolysaccharide (LPS) (Carlberg et al., 2013). Recent research has documented around 200 genes which are directly regulated by liganded VDR in human blood cells, and around 500 secondary targets (Hanel and Carlberg, 2022). The cells that appear to be the key targets of 1,25D regulation in the immune system are macrophages and dendritic cells. They have a high VDR expression level (Carlberg et al., 2013) and can produce 1,25D from its precursor 25D (Dusso et al., 1991). 1,25D directly regulates the expression of genes that are crucial to the functions of macrophages and dendritic cells, such as CD14 (Gombart et al., 2005; Carlberg, 2014b), cathelicidin (Liu et al., 2007), and TNFα (Cohen et al., 2001). However, the influence of 1,25D on the human innate immune response is complex because it has been shown that pre-treatment of human monocytes with 1,25D reduced their production of TNF-α and interleukin- (IL-) 6 in response to bacterial LPS (Zhang et al., 2012). Noteworthy, for around 700 genes regulated by VDR in human blood cells, the majority have been linked to downregulation of neutrophil degranulation, chemokine production, IFNγ-mediated signal transduction, and IL-6 production (Hanel and Carlberg, 2022).

When a virus infects the human body, the innate defenses are activated. Infected cells produce interferons *α* and *β*, which diffuse to neighboring cells to render them into an antiviral state. The virus-infected cells downregulate MHC class I molecules, whereby they are targets for killing by natural killer cells. Eventually, macrophages, using their toll-like receptors, may recognize the virus particles, phagocytose them, produce nitric oxide within phagolysosomes, and secrete defensins, cathelicidin, or TNFα into their environment. When innate responses are insufficient to eliminate the virus, adaptive immune mechanisms are activated. The most efficient process is the destruction of virus-infected cells by cytotoxic T lymphocytes, which recognize viral peptides that are presented at the cell surface of infected cells by MHC class I molecules. In addition, B lymphocytes may recognize certain patterns present in the viral envelope and then differentiate into plasma cells to produce a large number of antibodies. Both actions are under tight control by T helper cells (Delves et al., 2017). However, viral infections may lead to excessive immune responses, such as a cytokine storm (CS). This is a dangerous immune condition characterized by a release of about 150 inflammatory cytokines and mediators of inflammation (Delves et al., 2017). CS can lead to severe complications such as high fever, intravascular coagulation, tissue damage, multiple organ failure, and death (Wong et al., 2017). Walsh et al. examined how some of these life-threatening events may be attenuated by a sufficient level of 1,25D.

There are no unwanted side effects for taking vitamin D to ensure a proper level of 25D. Accordingly, Walsh et al. published their recommendations of “a vitamin D intake of 800–1000 IU per day, with a higher and monitored dose, e.g., 1,500–2000 IU per day, for vulnerable groups who have a confirmed or a likely low vitamin D status”. Whether vitamin D can provide an adjunct to therapy for patients who already have COVID-19 remains an open question. The data discussed by Tomaszewska et al. indicate that 25D (calcifediol), rather than vitamin D, should be used therapeutically.
